# Molecular investigation of *Coxiella burnetii* and *Francisella tularensis* infection in ticks in northern, western, and northwestern Iran

**DOI:** 10.1371/journal.pone.0289567

**Published:** 2023-08-17

**Authors:** Saber Esmaeili, Mina Latifian, Ahmad Mahmoudi, Ahmad Ghasemi, Ali Mohammadi, Alireza Mordadi, Seyyed Payman Ziapour, Saied Reza Naddaf, Ehsan Mostafavi

**Affiliations:** 1 National Reference Laboratory for Plague, Tularemia and Q Fever, Research Centre for Emerging and Reemerging Infectious Diseases, Pasteur Institute of Iran, Akanlu, KabudarAhang, Hamadan, Iran; 2 Department of Epidemiology and Biostatics, Research Centre for Emerging and Reemerging Infectious Diseases, Pasteur Institute of Iran, Tehran, Iran; 3 Department of Biology, Faculty of Science, Urmia University, Urmia, Iran; 4 Department of Microbiology, Research Center of Reference Health Laboratories, Ministry of Health and Medical Education, Tehran, Iran; 5 Department of Medical Entomology and Vector Control, School of Public Health and National Institute of Health Research, Tehran University of Medical Sciences, Tehran, Iran; 6 Department of Parasitology, Zoonoses, Research Center, Pasteur Institute of Iran, Amol, Mazandaran, Iran; 7 Department of Parasitology, Pasteur Institute of Iran, Tehran, Iran; University of Bari, ITALY

## Abstract

Tularemia and Q fever are endemic diseases in Iran; however, little information is available on the prevalence of the causative agents, *Coxiella burnetii* and *Francisella tularensis*, in Iranian ticks. This study investigated *C*. *burnetii* and *F*. *tularensis* among hard ticks in this country. We collected ticks from livestock and other mammals in Guilan, Mazandaran, Golestan (northern Iran), Kurdistan (western Iran), and West Azerbaijan (northwestern Iran) provinces. Genomic DNA from collected ticks was extracted and screened for *C*. *burnetii* and *F*. *tularensis* using Real-time PCR. A total of 4,197 ticks (belonging to 12 different species) were collected, and *Ixodes ricinus* (46.4%), *Rhipicephalus turanicus* (25%), and *Rhipicephalus sanguineus* sensu lato (19.1%) were the most collected species. Of 708 pooled tick samples, 11.3% and 7.20% were positive for *C*. *burnetii* and *F*. *tularensis*, respectively. The genus of *Rhipicephalus* had the highest (18.3%) *C*. *burnetii* infection among the collected tick pools (P<0.001). Furthermore, the most positive pools for *F*. *tularensis* belonged to *Haemaphysalis* spp. (44.4%). Kurdistan had the most significant percentage of *C*. *burnetii*-infected ticks (92.5%), and there was a meaningful relationship between the provinces and the infection (P< 0.001). The ticks from Golestan exhibited the highest *F*. *tularensis* infection rate (10. 9%), and the infection showed no significant relationship with the provinces (P = 0.19). Ticks collected from grasslands had a higher *Coxiella burnetii* infection rate than those collected from animals (39.4% vs. 7.9%; p<0.01). However, ticks collected from animal surfaces had a slightly higher rate of *Francisella tularensis* infection than those collected from grasslands (7.6% vs. 3.9%; p = 0.24). Here, we demonstrated the presence of both pathogens in the north (Guilan, Mazandaran, and Golestan provinces), the west (Kurdistan province), and the northwest (West Azerbaijan province) of Iran. The public health system should pay particular attention to tick bites in veterinary medicine and humans.

## Introduction

Zoonotic diseases have an increasing impact on global public health. More than 60% of the emerging infectious diseases in humans are zoonotic, and more than 70% have wildlife origin [1]. These infectious agents can be transmitted to humans through direct contact, aerosol inhalation, arthropod bites, and contaminated food and water consumption. Arthropods transmit about 25% of emerging zoonotic infectious diseases. Vector-borne diseases are considered a severe threat to human and animal health. The transmission of vector-borne diseases between humans and animals depends on a complex network of the interaction of different factors. These diseases mainly occur when the vectors, hosts, appropriate weather conditions, pathogens, and human populations exist at the same time [2–4].

Ticks are among the most significant vectors of arthropod-borne illnesses globally, capable of transmitting a broad range of infectious pathogens to people and animals. The epidemiology and ecology of tick-borne diseases are influenced by dynamic interactions between living and non-living factors. These include the biological characteristics of ticks and the associated pathogens, climate change, or changes related to human activities such as globalization, urbanization, travel, land-use change, habitat improvement, economics, politics, and demographic changes [5]. The awareness of the effects of tick-borne diseases is constantly increasing [6, 7]. Among tick-borne diseases, infections caused by *Coxiella burnetii* and *Francisella tularensis* have been reported in most regions of the world in recent years [8, 9].

*Coxiella burnetii* is a small gram-negative obligate intracellular highly infectious bacterium that causes Q fever. This pathogen is a particularly significant danger to people working with animals, such as slaughterhouse workers, farmers, or veterinarians, because of the rapid aerosol dispersal, survival in harsh environmental conditions, low infectious dosage, and high infectivity. It can be said that it is known as an occupational disease [8, 10, 11]. Domestic livestock is the main reservoir of *C*. *burnetii*. Q fever in animals is generally asymptomatic, and in pregnant domestic animals (cattel, sheep, and goats), it is associated with pneumonia and reproductive disorders such as abortion, stillbirth, placenta infection, uterine infection and infertility [12]. In the infectious cycle of *C*. *burnetii*, humans are considered accidental hosts for this zoonotic pathogen [13]. The main transmission route to humans is inhaling aerosols and dust particles contaminated with *C*. *burnetii* [14]. Tick bites, direct contact, consumption of raw milk and contaminated dairy products, blood transfusions, and sexual transmission are alternative routes for transmitting bacteria to individuals [15]. The manifestation of clinical features of Q fever in humans varies from asymptomatic to acute Q fever, chronic Q fever, and chronic fatigue syndrome. In humans, persistent acute and asymptomatic *C*. *burnetii* infections can proceed to a severe chronic form associated with endocarditis in 5–6% of cases [16]. The occurrence of Q fever endocarditis, if untreated, brings a significant mortality rate of up to 60% [17]. *C*. *burnetii* infection is widespread worldwide, with thousands of human clinical cases and positive animal and environmental cases documented yearly. According to reports in recent years, Q fever is considered an endemic zoonotic illness in Iran [18].

*Coxiella burnetii* is an endemic disease in Iran, and human cases of Q fever endocarditis have been identified in this country [19, 20]. In other studies, this pathogen has been identified in different parts of Iran in milk and abortion samples. In a systematic study, the prevalence of Q fever in cattel, goat, and sheep milk was reported as 15.1%, 7.8%, and 3.8%, respectively [21]. The prevalence of *C*. *burnetii* in abortion samples of domestic animals was reported as 24.7% in a study in different parts of Iran [22]. In another survey on slaughterhouse workers in Kerman province, considered risk groups for this disease, Q fever antibody was detected in 68% of the workers [23].

*Francisella tularensis* is a bacterium that causes tularemia, a highly infectious organism for humans and many animals, commonly in the Northern Hemisphere [9, 24]. This pathogenic agent comprises three subspecies, *tularensis*, *holarctica*, and *mediastica*. The infection by *F*. *tularensis* subsp. *tularensis* and *F*. *tularensis* subsp. *holarctica* can lead to tularemia in humans. *Francisella tularensis* has various animal reservoirs, including vertebrates and invertebrates [25]. Tularemia is a vector-borne infection transmitted to humans via the bites of infective ticks and fleas. Arthropods may acquire infection from infected animals and contaminated environmental water. Inhalation of contaminated aerosols, direct contact with the animal reservoir, arthropod (tick or deer-fly) bites, and consumption of contaminated water are the primary modes of transmission of tularemia infection to humans [9]. Tularemia clinical features can vary from asymptomatic to severe cases leading to human death [24]. The first human clinical case of tularemia in Iran was reported in Kurdistan, West of Iran 1981 [26]. In recent years, positive serological and molecular cases in rodents and humans have been reported from different regions of Iran, indicating the endemicity of this disease in Iran [27–32].

Considering the limited data on the *C*. *burnetii* and *F*. *tularensis* infections among ticks collected from vegetation, livestock, and small mammals in Iran, the present study aimed to investigate the status of infections with these two pathogenic agents among ticks and spleen samples of small mammals in the north, west, and northwest of Iran.

## Material and methods

### Ethics approval and consent to participate

The study complied with the Research Ethical Committee (REC) guidelines for experimental and clinical studies at the Pasteur Institute of Iran (IR.PII.REC.1395.29). Tick and spleen samples were collected from small mammals according to the REC protocol at the Pasteur Institute of Iran.

### Study area

**Fig 1:**
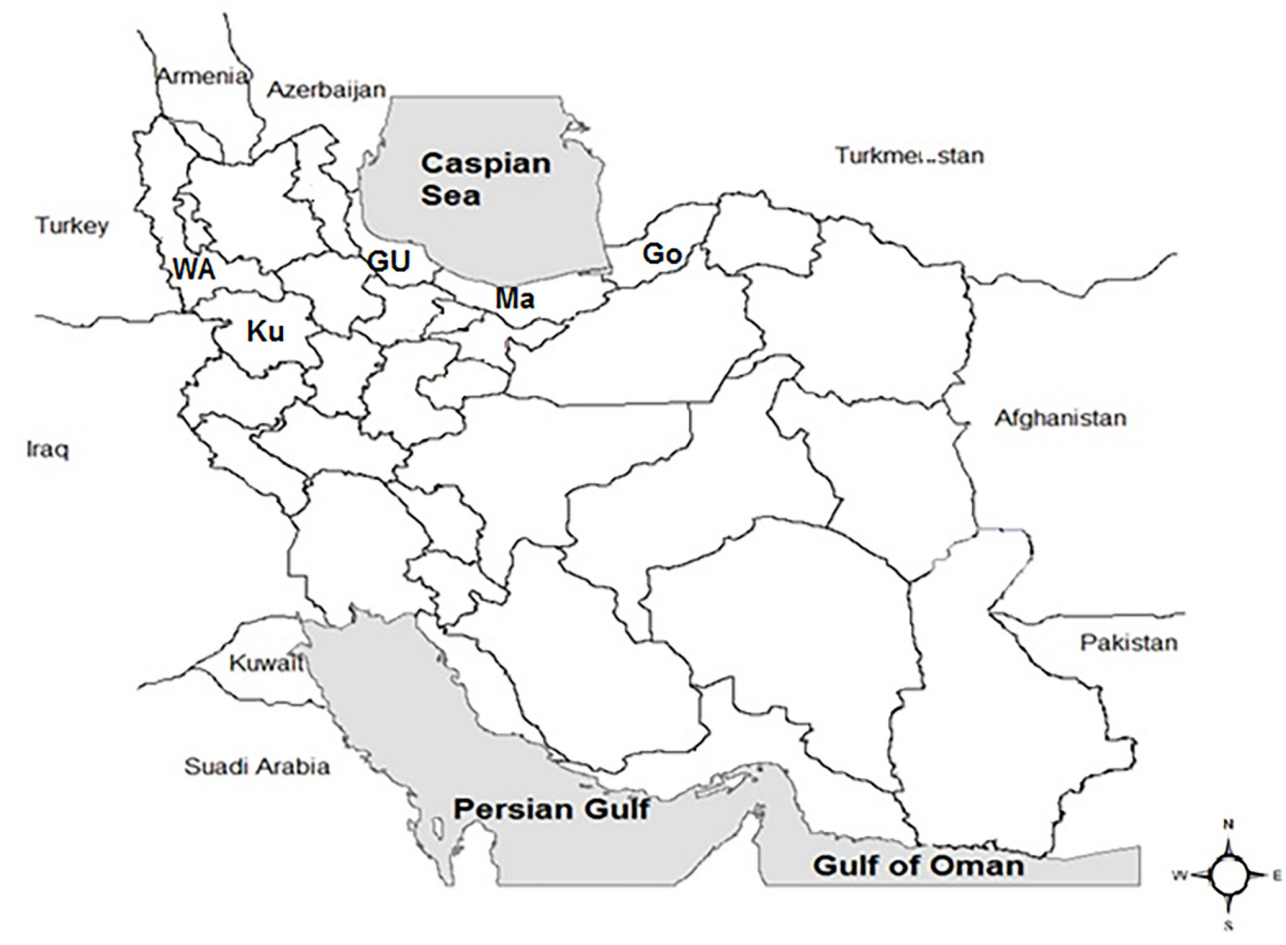
Provinces in which ticks were collected in this study. Abbreviations: Guilan (Gu), Mazandaran (Ma), Golestan (Go), Kurdistan (Ku), and West Azerbaijan (WA).

The study area in Iran included Guilan, Mazandaran, and Golestan provinces in the north, Kurdistan in the west, and West Azerbaijan in the northwest (Fig 1). The north of Iran extends from the distance south of the Caspian Sea to the north of the Alborz mountains. The area of the north of the country is 58,167 Km^2^, and its population is more than 10 million people. This region has a moderate and humid climate, and agriculture and livestock farming are practised in the rural areas. The province of Kurdistan is in the west of Iran and adjacent to the country of Iraq. This province occupies 29,500 Km^2^ and has a population of ~2 million. The climate of this region is hot and humid Mediterranean, and it is one of the important agriculture and animal husbandry zones. West Azerbaijan province is located in the northwest of Iran and borders with Turkey. The area of this province is 3,700 Km^2^, and its population is about 4 million people. This province is mountainous, with many rivers suitable for agriculture and animal husbandry. This province has mild weather in spring and summer and cold and snowy in winter.

### Sample collection

The sampling in this study was conducted from 23 October to 6 November 2017 and from 8 to 22 January 2018. The ticks from domestic livestock (sheep, goats, cattels, camels, horses, dogs, and donkeys) and other mammals (rodents and hedgehogs) were collected. Besides, the blanketing method was used to collect ticks from grasslands. The collected ticks were identified based on available morphological keys [33], labelled, and stored in 70% alcohol at 4°C until DNA extraction.

### Small mammal trapping

Hand-made wooden 25×15×15 cm^3^ life traps were used to entrap small mammals. This type of trap is suitable for capturing all small mammals in the study area. The traps were placed in plains, and dates, lettuce, and cucumber were used as bait. The spleen samples of the trapped small mammals were collected after identifying them with morphological keys [34] and kept at -20° C until DNA extraction.

### DNA extraction from ticks

After the identification of collected ticks, the ticks were pooled for DNA extraction. 4,197 collected ticks were pooled based on the same tick’s species, the same collected locations, the same host, the same tick sex, and the growth stage of ticks. Finally, the pools of ticks included 1 to 22 ticks based on the above criteria and 708 pools were prepared. The 708 pools were first homogenized in liquid nitrogen and sterile PBS, and the DNA was extracted by the potassium acetate method recommended by Rodríguez et al. [35]. Amount of 500 μl of lysis buffer (0.1 M TRIS-HCl, 0.05 M EDTA, 0.2 M sucrose, and 0.5% SDS) with 10 mL proteinase K were added to the homogenized specimens and incubated at 56°C overnight. Then, 120 μl 5 M sodium acetate was added to the specimens and kept on ice for 10 min. The suspensions were centrifuged at 12,000 ×g for 10 min, and the supernatant was recovered. For precipitation of DNA, 35 μl of 4 M sodium acetate and 1 ml of pure ethanol were added to specimens, mixed well, and kept on ice for 10 min. The samples were centrifuged at 12,000 ×g for 20 min, and the supernatant was discarded. The precipitates were washed with 500 μl of 70% ethanol, and the remaining alcohol was allowed to dry completely at room temperature. Finally, completely-dried precipitates were dissolved in 200 μl of elution buffer (1 molar Tris-HCl, 1 molar EDTA) and kept at -20°C until the analysis.

### DNA extraction from spleen samples of small mammals

DNA extraction of small mammals (hedgehogs, shrews, and rodents) spleen samples was performed using a commercial High Pure PCR Template Preparation kit (Roche, Germany). Approximately 200 μl of lysis buffer and 40 μl of proteinase K were added to 25–50 mg samples and incubated at 55°C overnight. Then, 200 μl of binding buffer was added to each sample and incubated at 70°C for 25 min. Following adding 100 μl of isopropanol to the extraction columns, the suspensions were centrifuged at 8,000 ×g for one minute. Then, 500 μl of inhibitor buffer was added to each column and centrifuged for one minute at 8,000 ×g. In the next stage, 500 μl of wash buffer was added to each column, centrifuged at 12,000 ×g for one min, and then the wash buffer step was repeated. Finally, 120 μl of elution buffer solution was added to the samples and DNAs were extracted. Extracted DNAs were kept at—20°C until the molecular test.

### Detection of *Coxiella burnetii* and *Francisella tularensis*

Extracted DNA from ticks and the spleen of small mammals were screened for *C*. *burnetii* and *F*. *tularensis* by a real-time PCR using the specific primers and the probe specific for the gene IS*1111* and ISftu2 genes respectively. The probes were marked with 6-Carboxyfluorescein (6-FAM) fluorescent dye as a reporter dye and TAMRA as a quencher. The 20 μl reactions contained 10 μl commercial 2x RealQ Plus Master Mix (Ampliqon, Denmark), 900 nmol of forward primers (**5**’**-**AAAACGGATAAAAAGAGTCTGTGGTT**-3**’ for *C*. *burnetii* and **5**’**-**TTGGTAGATCAGTTGGTAGGATAACC**-3**’ for *F*. *tularensis*), 900 nmol reverse primers (**5**’**-**CCACACAAGCGCGATTCAT**-3**’ for *C*. *burnetii* and **5**’**-** TGAGTTTTATCCTCTGACAACAATATTTC**-3**’ for *F*. *tularensis*), 200 nmol of the probes (**5**’**-**6-FAM-AAAGCACTCATTGAGCGCCGCG-TAMRA**-3**’ for *C*. *burnetii* and **5**’**-**6-FAM-AAAATCCATGCTATGACTGATGCTTTAGGTAATCCA- TAMRA**-3’** for *F*. *tularensis*), and 4 μl template DNA and distilled water to the final volume. *Coxiella burnetii* strain Nine Mile RSA493 and *F*. *tularensis* subsp. *holarctica* NCTC 10857 was used as a positive control for the detection of *C*. *burnetii* and *F*. *tularensis* in real-time PCR tests, respectively. Also, distilled water was used as the negative control. The amplification was performed in a Corbett 6000 Rotor-Gene system thermocycler (Corbett, Victoria, Australia) programmed for 10 min at 95°C and 45 cycles at 95°C for 15 sec and 60°C for 60 seconds. The reading in each cycle was performed in the green spectrum at 60°C. Rotor-Gene Q Series Software was used for analyzed the real-time PCR results.

### Statistical analysis

The data were analyzed by SPSS software (version 16). Chi-squared, Fisher exact, and logistic regression tests were used to compare the variables. A P-value <0.05 was considered statistically significant. Statistical analysis explored the correlation between the incidence of examined diseases and factors like tick species, collection province, and tick-animal host relationship.

## Results

### Tick identification

A total of 4,197 ticks were collected in the present study. Among the collected ticks, 56.1% (n = 2356) belonged to Mazandaran province, 34.7% (n = 1456) to Golestan province, 4.4% (n = 187) to Guilan province, 2.7% (n = 115) to Kurdistan province, and 1.9% (n = 83) to West Azerbaijan province. Among the ticks, 36.4% (n = 1530) were male, 62.5% (n = 2624) were female, and 1% (n = 43) were nymphs.

Twelve tick species belonging to 4 different genera (*Ixodes*, *Haemaphysalis*, *Hyalomma*, and *Rhipicephalus*) were identified, and *I*. *ricinus* (46.4%), *Rh*. *turanicus* (25%), *Rh*. *sanguineus* sensu lato (19.1%), *Hy*. *marginatum* (3.4%), and *Rh*. *bursa* (2.8%) species were the most collected ticks (Table 1).

**Table 1:** The tick species collected from the studied provinces in 2017–2018.

Genus	Species	Mazandaran N (%)	Golestan N (%)	Guilan N (%)	Kurdistan N (%)	West-Azerbaijan N (%)	Total N (%)
*Ixodes*	*I*. *ricinus*	1805 (76.6)	6 (0.4)	140 (74.9)	-	-	1951 (46.4)
*Haemaphysalis*	*Ha*. *concinna*	6 (0.2)	-	1 (0.5)	-	-	7 (0.2)
	*Ha*. *Inermis*	3 (0.1)	-	1 (0.5)	-	-	4 (0.1)
	*Ha*. *punctata*	-	-	1 (0.5)	-	-	1 (0.02)
*Hyalomma*	*Hy*. *anatolicum*	-	40 (2.7)	-	-	4 (4.8)	44 (1.00)
	*Hy*. *marginatum*	56 (2.4)	65 (4.5)	16 (8.6)	-	6 (7.2)	143 (3.4)
	*Hy*. *dromedarii*	-	18 (1.2)	-	-	-	18 (0.4)
	*Hyalomma* spp.	-	-	-	-	3 (3.6)	3 (0.1)
*Rhipicephalus*	*Rh*. *bursa*	48 (2.0)	1 (0.1)	-	-	70 (84.3)	119 (2.8)
	*Rh*. *sanguineus*	343 (14.5)	343 (23.6)	1 (0.5)	115 (100.0)	-	802 (19.1)
	*Rh*. *turanicus*	61 (2.6)	983 (67.5)	9 (4.8)	-	-	1053 (25.1)
	*Rh*. *annulatus*	34 (1.4)	-	18 (9.6)	-	-	52 (1.2)
**Total**		2356 (100)	1456 (100)	187 (100)	115 (100)	83 (100)	4197 (100)

Of the collected ticks, 45.46% (n = 1908) were collected from cattel, 34.7% (n = 1458) from sheep, 9.33% (n = 384) from goats, 0.11% (n = 5) from horses, 0.04% (n = 2) from donkeys, 3.0% (n = 128) from camels, 1.19% (n = 50) from dogs, and 1.1% (n = 47) from hedgehogs.

### *Coxiella burnetii* detection

Of the 708 pooled tick samples, 80 (11.3%) were positive for *C*. *burnetii*. The prevalence of *C*. *burnetii* in *Rhipicephalus* (18.3%) was significantly higher compared to other ticks genera (*P*<0.001). Among tick species, the highest prevalence belonged to *Rh*. *sanguineus* sensu lato (26.1%) and *Rh*. *turanicus* (15.2%) (Table 2). About 13.8% of positive pools belonged to Golestan province, 5.82% to Mazandaran province, 92.5% to Kurdistan province, and 11% to West Azerbaijan province. There was a significant relationship between *C*. *burnetii* infection and the provinces (*P*<0.001). The highest positive pools were from Sanandaj county, in Kurdistan province, i.e., all 14 pools from this area were positive. Bandar Torkaman county showed the highest number of positive pools in Golestan province; out of 56 pools, 13 were reported positive. Amol County in Mazandaran province showed the highest positive rate, with 10 out of 78 pools being positive. In Guilan province, no ticks were positive for *C*. *burnetii* (Table 3). The highest number of positive pools according to the hosts from which the ticks were collected belonged to *Rh*. *turanicus* and *Rh*. *sanguineus* collected from sheep in Golestan province, where 17 (25%) and 8 (19%) pools out of 80 pools were reported positive. Also, it should be mentioned that 92.6% (N = 25) of *Rh*. *sanguineus* collected from the grassland in Kurdistan province were infected with this pathogen (Table 4).

**Table 2:** *Coxiella burnetii* and *Francisella tularensis* infections among ticks in Iran (2017–2018).

Genus	Species	Number of tested pools	Number of positive pools *for F*. *tularensis* in species (%)	Number of positive pools *for F*. *tularensis* per genus (%)	p-value	Number of positive pools for *C*. *burnetii* (%)	Number of positive pools for C. burnetii per genus (%)	p-value
***Ixodes***	***I*. *ricinus***	259	7 (2.7)	7(2.7)	<0.001	8 (3.1)	8 (3.1)	<0.001
***Haemaphysalis***	***Ha*. *concinna***	4	0 (0)	4(44.4)		0 (0)	0 (0)	
	***Ha*. *inermis***	4	4 (100)			0 (0)		
	***Ha*. *punctata***	1	0 (0)			0 (0)		
***Hyalomma***	***Hy*. *anatolicum***	13	1 (7.7)	4(5.8)		1 (7.7)	4 (5.8)	
	***Hy*. *marginatum***	49	3 (6.1)			3 (6.1)		
	***Hy*. *dromedarii***	4	0 (0)			0 (0)		
	***Hyalomma* spp.**	3	0 (0)			0 (0)		
***Rhipicephalus***	***Rh*. *bursa***	45	2 (4.4)	36(9.70)		3 (6.7)	68 (18.3)	
	***Rh*. *sanguineus***	172	18 (10.5)			45 (26.2)		
	***Rh*. *turanicus***	131	15 (11.4)			20 (15.3)		
	***Rh*. *annulatus***	23	1 (4.3)			0 (0)		
	**Total**	708	51 (7.2)			80 (11.3)		

**Table 3:** Prevalence of *Coxiella burnetii* and *Francisella tularensis* in ticks based on localities.

Counties	Provinces	Number of pools tested	Number of positive pools for *C*. *burnetii* per genus (%)	Number of positive pools for *F*. *tularensis* per genus
				(%)
**Golestan**	Aq Qala	67	0 (0)	7 (10.4)
	Bandar Torkaman	56	13 (23.2)	5 (8.9)
	Aliabad	8	1 (12.5)	2 (25)
	Gorgan	39	5 (12.8)	5 (12.8)
	Gomishan	8	0 (0)	1 (12.5)
	Aliabad-e-Katul	23	9 (39.1)	2 (8.7)
	Gonbad Kavus	1	0(0)	0 (0)
	**Total**	202	28 (13.8)	22 (10. 9)
**Mazandaran**	Nur	62	4 (6.45)	5 (8)
	Amol	78	10 (12.8)	8 (10.3)
	Babol	55	0 (0)	1 (1.8)
	Sari	71	5 (7.04)	3 (4.2)
	Qaemshahr	55	2 (3.6)	5 (9)
	Mahmudabad	16	1 (6.2)	0 (0)
	Savadkuh	41	0 (0)	1 (2.4)
	**Total**	378	22 (5.8)	23 (6.1)
**Guilan**	Talesh	46	0 (0)	3 (6.5)
	Masal	5	0 (0)	0 (0)
	Rudsar	1	0 (0)	0 (0)
	Lahijan	6	0 (0)	0 (0)
	**Total**	58	0 (0)	3 (5.2)
**Kurdistan**	Sanandaj	14	14 (100)	1 (7.1)
	Divandarreh	9	8 (88.8)	0 (0)
	Mariwan	4	3 (75)	0 (0)
	**Total**	27	25 (92.5)	1 (3.7)
**West-Azerbaijan**	Showt	43	5 (11.6)	2 (4.6)
	**Total**	43	5 (11.6)	2 (4.6)

**Table 4:** Prevalence of *Coxiella burnetii* and *Francisella tularensis* in ticks according to the host and provinces studied.

Host	Tick species	Number tested (% positive) for *Coxiella burnetii*						Number tested (%positive) for *Francisella tularensis*					
		Maz. N (%)	Gol. N (%)	Guil. N (%)	Kurd. N (%)	Azer. N (%)	Total N (%)	Maz. N (%)	Gol. N (%)	Guil. N (%)	Kurd. N (%)	Azer. N (%)	Total N (%)
**Cattel**	*I*. *ricinus*	179 (1.7)	0(0)	32 (0)	0(0)	0(0)	211 (1.4)	179 (1.2)	0(0)	32 (3.1)	0 (0)	0 (0)	211 (1.4)
	*Hy*. *Marginatum*	4 (0)	6 (0)	4 (0)	0(0)	0(0)	14 (0)	4 (25)	6 (0)	4 (0)	0 (0)	0 (0)	14 (7.1)
	*Rh*. *Turanicus*	4 (0)	5 (0)	1 (0)	0(0)	0(0)	10 (0)	4 (0)	5 (20)	1 (0)	0 (0)	0 (0)	10 (10)
	*Ha*. *Inermis*	1 (0)	0(0)	1 (0)	0(0)	0(0)	2 (0)	1 (100)	0 (0)	1 (100)	0 (0)	0 (0)	2 (100)
	*Ha*. *Concinna*	3 (0)	0(0)	1 (0)	0(0)	0(0)	4 (0)	3 (0)	0 (0)	1 (0)	0 (0)	0 (0)	4 (0)
	*Rh*. *Annulatus*	9 (0)	0(0)	14 (0)	0(0)	0(0)	23 (0)	9 (0)	0 (0)	14 (7.1)	0 (0)	0 (0)	23 (4.3)
	*Rh*. *Sanguineus*	5 (20)	5 (0)	1 (0)	0(0)	0(0)	11 (9)	5 (40)	5 (20)	0 (0)	0 (0)	0 (0)	11 (27.3)
	*Hy*. *Anatolicum*	0(0)	1 (100)	0(0)	0(0)	0(0)	1 (100)	0 (0)	1 (100)	0 (0)	0 (0)	0 (0)	1 (100)
	Total	205 (1.9)	17 (5.9)	54 (0)	0(0)	0(0)	276 (1.8)	205 (2.9)	17 (17.6)	54 (5.5)	0 (0)	0 (0)	276 (4.3)
**Camel**	*Hy*. *Anatolicum*	0 (0)	9 (0)	0 (0)	0 (0)	0 (0)	9 (0)	0 (0)	9 (0)	0 (0)	0 (0)	0 (0)	9 (0)
	*Hy*. *Marginatum*	0 (0)	8 (0)	0 (0)	0 (0)	0 (0)	8 (0)	0 (0)	8 (12.5)	0 (0)	0 (0)	0 (0)	8 (12.5)
	*Rh*. *Sanguineus*	0 (0)	1 (0)	0 (0)	0 (0)	0 (0)	1 (0)	0 (0)	1 (0)	0 (0)	0 (0)	0 (0)	1 (0)
	*Rh*. *Turanicus*	0 (0)	4 (0)	0 (0)	0 (0)	0 (0)	4 (0)	0 (0)	4 (25)	0 (0)	0 (0)	0 (0)	4 (25)
	*Hy*. *Dromedarii*	0 (0)	4 (0)	0 (0)	0 (0)	0 (0)	4 (0)	0 (0)	4 (0)	0 (0)	0 (0)	0 (0)	4 (0)
	Total	0 (0)	26 (0)	0 (0)	0 (0)	0 (0)	26 (0)	0 (0)	26 (7.7)	0 (0)	0 (0)	0 (0)	26 (7.7)
**Dog**	*Rh*. *Sanguineus*	0 (0)	3 (0)	0 (0)	0 (0)	0 (0)	3 (0)	0 (0)	3 (33.3)	0 (0)	0 (0)	0 (0)	3 (33.3)
	*Rh*. *Turanicus*	5 (0)	9 (0)	0 (0)	0 (0)	0 (0)	14 (0)	5 (20)	9 (0)	0 (0)	0 (0)	0 (0)	14 (7.1)
	*I*. *ricinus*	1 (0)	0 (0)	0 (0)	0 (0)	0 (0)	1 (0)	1 (0)	0 (0)	0 (0)	0 (0)	0 (0)	1(0)
	Total	6 (0)	12 (0)	0 (0)	0 (0)	0 (0)	18 (0)	6 (16.6)	12 (8.3)	0 (0)	0 (0)	0 (0)	18 (11.1)
**Donkey**	*Rh*. *Sanguineus*	0 (0)	1 (0)	0 (0)	0 (0)	0 (0)	1 (0)	0 (0)	1 (0)	0 (0)	0 (0)	0 (0)	1 (0)
	Total	0 (0)	1 (0)	0 (0)	0 (0)	0 (0)	1 (0)	0 (0)	1 (0)	0 (0)	0 (0)	0 (0)	1 (0)
**Goat**	*Ha*. *Punctata*	0 (0)	0 (0)	1 (0)	0 (0)	0 (0)	1 (0)	0 (0)	0 (0)	1 (0)	0 (0)	0 (0)	1 (0)
	*Rh*. *Sanguineus*	31 (16.1)	14 (0)	0 (0)	0 (0)	0 (0)	45 (11.1)	31 (9.7)	14 (7.1)	0 (0)	0 (0)	0 (0)	45 (8.9)
	*Rh*. *Turanicus*	4 (0)	15 (6.7)	0 (0)	0 (0)	0 (0)	19 (5.3)	4 (25)	15 (6.7)	0 (0)	0 (0)	0 (0)	19 (10.5)
	*I*. *ricinus*	10 (20)	0 (0)	0 (0)	0 (0)	0 (0)	10 (20)	10 (20)	0 (0)	0 (0)	0 (0)	0 (0)	10 (20)
	*Hy*. *Marginatum*	9 (0)	0 (0)	0 (0)	0 (0)	0 (0)	9 (0)	9 (0)	0 (0)	0 (0)	0 (0)	0 (0)	9 (0)
	*Rh*. *Bursa*	6 (0)	0 (0)	0 (0)	0 (0)	0 (0)	6 (0)	6 (0)	0 (0)	0 (0)	0 (0)	0 (0)	6 (0)
	*Ha*. *Inermis*	2 (0)	0 (0)	0 (0)	0 (0)	0 (0)	2 (0)	2 (100)	0 (0)	0 (0)	0 (0)	0 (0)	2 (100)
	Total	62 (11.3)	29 (3.4)	1 (0)	0 (0)	0 (0)	92 (8.7)	62 (12.9)	29 (6.9)	1 (0)	0 (0)	0 (0)	92 (10.9)
**Sheep**	*Rh*. *Sanguineus*	42 (14.3)	42 (19)	0 (0)	0 (0)	0 (0)	84 (16.7)	42 (7.1)	42 (14.3)	0 (0)	0 (0)	0 (0)	84 (11)
	*Rh*. *Turanicus*	12 (8.3)	68 (25)	0 (0)	0 (0)	0 (0)	80 (22.5)	12 (25)	68 (10.3)	0 (0)	0 (0)	0 (0)	80 (12.5)
	*I*. *ricinus*	36 (8.3_)	1 (0)	0 (0)	0 (0)	0 (0)	37 (8.1)	36 (2.8)	1 (100)	0 (0)	0 (0)	0 (0)	37 (5.4)
	*Rh*. *Bursa*	4 (0)	1 (0)	0 (0)	0 (0)	0 (0)	5 (0)	4 (0)	1 (0)	0 (0)	0 (0)	0 (0)	5 (0)
	*Hy*. *Marginatum*	8 (12.5)	0 (0)	0 (0)	0 (0)	0 (0)	8 (12.5)	8 (12.5)	0 (0)	0 (0)	0 (0)	0 (0)	8(12.5)
	Total	102 (10.8)	112 (22.3)	0 (0)	0 (0)	0 (0)	214 (16.8)	102 (7.8)	112 (12.5)	0 (0)	0 (0)	0 (0)	214 (10.3)
**Hedgehog**	*Rh*. *Turanicus*	0 (0)	4 (25)	0 (0)	0 (0)	0 (0)	4 (25)	0 (0)	4 (0)	0 (0)	0 (0)	0 (0)	4 (0)
	Total	0 (0)	4 (25)	0 (0)	0 (0)	0 (0)	4 (25)	0 (0)	4 (0)	0 (0)	0 (0)	0 (0)	4 (0)
**Horse**	*Hy*. *Marginatum*	0 (0)	1 (0)	0 (0)	0 (0)	0 (0)	1 (0)	0 (0)	1 (0)	0 (0)	0 (0)	0 (0)	1 (0)
	Total	0 (0)	1 (0)	0 (0)	0 (0)	0 (0)	1 (0)	0 (0)	1 (0)	0 (0)	0 (0)	0 (0)	1 (0)
**All animals**	*I*. *ricinus*	226 (3.5)	1 (0)	32 (0)	0 (0)	0 (0)	259 (3.1)	226 (2.2)	1 (100)	32 (3.1)	0 (0)	0 (0)	259 (2.7)
	*Ha*. *Concinna*	3 (0)	0 (0)	1 (0)	0 (0)	0 (0)	4 (0)	3(0)	0 (0)	1 (0)	0 (0)	0 (0)	4 (0)
	*Ha*. *Inermis*	3 (0)	0 (0)	1 (0)	0 (0)	0 (0)	4 (0)	3 (100)	0 (0)	1 (100)	0 (0)	0 (0)	4 (100)
	*Ha*. *Punctata*	0 (0)	0 (0)	1 (0)	0 (0)	0 (0)	1 (0)	0 (0)	0 (0)	1 (0)	0 (0)	0 (0)	1 (0)
	*Hy*. *Anatolicum*	0 (0)	10 (10)	0 (0)	0 (0)	0 (0)	10 (10)	0 (0)	10 (10)	0 (0)	0 (0)	0 (0)	10 (10)
	*Hy*. *Marginatum*	21 (4.8)	15 (0)	4 (0)	0 (0)	0 (0)	40 (2.5)	21 (9.5)	15 (6.7)	4 (0)	0 (0)	0 (0)	40 (7.5)
	*Hy*. *Dromedarii*	0 (0)	4 (0)	0 (0)	0 (0)	0 (0)	4 (0)	0 (0)	4 (0)	0 (0)	0 (0)	0 (0)	4 (0)
	*Hyalomma spp*.	0 (0)	0 (0)	0 (0)	0 (0)	0 (0)	0 (0)	0 (0)	0 (0)	0 (0)	0 (0)	0 (0)	0 (0)
	*Rh*. *Bursa*	10 (0)	1 (0)	0 (0)	0 (0)	0 (0)	11 (0)	10 (0)	1 (0)	0 (0)	0 (0)	0 (0)	11 (0)
	*Rh*. *Sanguineus*	78 (15.3)	66 (12.1)	1 (0)	0 (0)	0 (0)	145 (13.8)	78 (12.3)	66 (13.6)	1 (0)	0 (0)	0 (0)	145 (11.7)
	*Rh*. *Turanicus*	24 (4.1)	105 (18.1)	1 (0)	0 (0)	0 (0)	131 (15.3)	24 (20.8)	105 (9.5)	1 (0)	0 (0)	0 (0)	131 (11.4)
	*Rh*. *Annulatus*	9 (0)	0 (0)	14 (0)	0 (0)	0 (0)	23 (0)	9 (0)	0 (0)	14 (7.1)	0 (0)	0 (0)	23 (4.3)
	Total	375 (5.9)	202 (13.9)	55 (0)	0 (0)	0 (0)	632 (7.9)	375 (6.1)	202 (10.9)	55 (5.4)	0 (0)	0 (0)	632 (7.6)
**Grassland**	*Rh*. *Sanguineus*	0 (0)	0 (0)	0 (0)	27 (92.6)	0 (0)	27 (92.6)	0 (0)	0 (0)	0 (0)	27 (3.7)	0 (0)	27 (3.7)
	*Rh*. *Bursa*	2 (0)	0 (0)	0 (0)	0 (0)	32 (9.4)	34 (8.8)	2 (0)	0 (0)	0 (0)	0 (0)	32 (6.3)	34 (5.9)
	*Hyalomma spp*.	0 (0)	0 (0)	0 (0)	0 (0)	3 (0)	3 (0)	0 (0)	0 (0)	0 (0)	0 (0)	3 (0)	3 (0)
	*Hy*. *Marginatum*	1 (0)	0 (0)	3 (0)	0 (0)	5 (40)	9 (22.2)	1 (0)	0 (0)	3 (0)	0 (0)	5 (0)	9 (0)
	*Hy*. *Anatolicum*	0 (0)	0 (0)	0 (0)	0 (0)	3 (0)	3 (0)	0 (0)	0 (0)	0 (0)	0 (0)	3 (0)	3 (0)
	Total	3 (0)	0 (0)	3 (0)	27/ (92.6)	43 (11.6)	76 (39.4)	3 (0)	0 (0)	3 (0)	27 (3.7)	43 (4.7)	76 (3.9)

The rate of *C*. *burnetii* infection in ticks was influenced by the type of host animal, with the highest infection rates observed in collected ticks from the body surface of sheep (16.8%), goats (8.7%), and cattel (1.8%) (p<0.01). Ticks collected from grasslands were significantly more infected with *C*. *burnetii* compared to those collected from animal surfaces (39.4% vs. 7.9%; p<0.01) (Table 4).

### *Francisella tularensis* detection

Out of 708 pools, 51 (7.2%) were reported positive for *F*. *tularensis*. Based on the test results, the genus *Haemaphysalis* had the highest *F*. *tularensis* infection among the collected ticks, and 4 out of 9 pools were reported positive for *F*. *tularensis*. There was a significant relationship between *F*. *tularensis* infection and the ticks genus (*P*<0.001); the genera *Haemaphysalis* (44.4%) and *Rhipicephalus* (9.7%) had the highest *F*. *tularensis* infection. Among the tick species, the highest prevalence belonged to *H*. *inermis* (100%), *Rh*. *turanicus* (11.4%), and *Rh*. *sanguineus* sensu lato (10.4%) (Table 2). Based on the results, 10.89% of positive pools belonged to Golestan Province, 6.1% to Mazandaran Province, 5.2% to Guilan Province, 3.7% to Kurdistan Province, and 4.6% to West Azerbaijan Province, respectively. However, there was no significant relationship between *F*. *tularensis* and different provinces (*P* = 0.19). The highest positive pools of *F*. *tularensis* were reported in Gorgan county of Golestan province, and 5 out of 39 pools were positive. In Mazandaran province, the highest positive pool was seen in Amol County, where 8 out of 78 pools were positive. In Talesh County of Guilan province, 3 out of 46 pools were positive. In Sanandaj county of Kurdistan province, 1 out of 14 pools was positive, and 2 out of 43 pools in West Azerbaijan province were positive (Table 3).

Ticks collected from the body surface of dogs (11.1%), goats (10.9%), and sheep (10.3%) had the highest infection rates for *F*. *tularensis*, but there was no statistically significant difference between the level of *Francisella* infection and the type of host animal (p = 0.28). The rate of *F*. *tularensis* infection in ticks collected from grasslands (3.9%) did not differ significantly from those collected from animal surfaces (7.6%) (p = 0.24).

### *Coxiella burnetii* and *Francisella*. *tularensis* in animals

Of 135 spleen DNAs belonging to small mammals (Table 5), none exhibited *C*. *burnetii* or *F*. *tularensis* infections.

**Table 5:** Geographic distribution of the included small mammals in the present study.

Species/ Province	Mazandaran N (%)	Guilan N (%)	Golestan N (%)	Kurdistan N (%)	West-Azerbaijan N (%)	Total
*Apodemus hyrcanicus*	2 (11.1)	2 (16.6)	-	0(0)	0(0)	4 (3.0)
*Mus musculus*	2 (11.1)	4 (33.3)	1 (7.14)	0(0)	0(0)	7 (5.2)
*Rattus norvegicus*	13 (72.2)	0(0)	0(0)	0(0)	0(0)	13 (9.6)
*Rattus rattus*	1 (5.5)	4 (33.3)	0(0)	0(0)	0(0)	5 (3.7)
*Crocidura caspica*	0(0)	1 (8.3)	0(0)	0(0)	0(0)	1 (0.7)
*Microtus obscurus*	0(0)	1 (8.3)	0(0)	0(0)	0(0)	1 (0.7)
*Nesokia indica*	0(0)	0(0)	6 (42.8)	0(0)	0(0)	6 (4.4)
*Erinaceus concolor*	0(0)	0(0)	3 (21.4)	0(0)	0(0)	3 (2.2)
*Apodemus uralensis*	0(0)	0(0)	1 (7.14)	0(0)	0(0)	1 (0.7)
*Crocidura suaveolens*	0(0)	0(0)	1 (7.14)	0(0)	0(0)	1 (0.7)
*Microtus paradoxus*	0(0)	0(0)	2 (14.2)	0(0)	0(0)	2 (1.48)
*Apodemus witherbyi*	0(0)	0(0)	0(0)	22 (26.8)	0(0)	22 (16.3)
*Apodemus ponticus*	0(0)	0(0)	0(0)	1 (1.2)	0(0)	1 (0.7)
*Arvicola persicus*	0(0)	0(0)	0(0)	8 (9.7)	0(0)	8 (5.9)
*Nothocricetulus migratorius*	0(0)	0(0)	0(0)	2 (2.4)	0(0)	2 (1.5)
*Meriones persicus*	0(0)	0(0)	0(0)	5 (6.1)	1 (11.1)	6 (4.4)
*Meriones tristrami*	0(0)	0(0)	0(0)	1 (1.2)	0(0)	1 (0.7)
*Microtus schidlovskii*	0(0)	0(0)	0(0)	2 (2.4)	0(0)	2 (1.5)
*Microtus qazvinensis*	0(0)	0(0)	0(0)	4 (4.9)	0(0)	4 (3.0)
*Mus macedonicus*	0(0)	0(0)	0(0)	2 (2.4)	0(0)	2 (3.0)
*Microtus qazvinensis*	0(0)	0(0)	0(0)	35 (42.7)	0(0)	35 (25.9)
*Meriones vinogradovi*	0(0)	0(0)	0(0)	0(0)	7 (77.7)	7 (5.2)
*Microtus socialis*	0(0)	0(0)	0(0)	0(0)	1 (11.1)	1 (0.7)
Total	18 (100)	12 (100)	14 (100)	82 (100)	9 (100)	135 (100)

## Discussion

The present study investigated the hard ticks from three Northern provinces (Mazandaran, Golestan, and Guilan) and the northwestern and western provinces of Iran (Kurdistan, West Azerbaijan) for *C*. *burnetii* and *F*. *tularensis*. Our real-time PCR showed that 11.3% of tick pools were positive for *C*. *burnetii* and 7.2% for *F*. *tularensis*, confirming the circulation of both pathogens via the ticks in the regions.

Due to the spread of tick-borne diseases, including the two pathogens in the present study in the Middle East, more studies should be conducted. Also, the possibility of human bites by ticks as vectors of these diseases and the detection of human cases infected with these two pathogens in Iran [19, 20] requires a broad approach to determine the presence of these bacteria in ticks and animals (mammals).

In this study, *C*. *burnetii* DNA was identified in collected ticks from Northern provinces (except for Guilan), Kurdistan, and West Azerbaijan provinces, and the highest positive pools belonged to Kurdistan province. Domestic ruminants are considered the main reservoirs for *C*. *burnetii*, but it seems that ticks may also be involved in the cycle of transmission and maintenance of this pathogen [36]. The first tick species in which *C*. *burnetii* was identified belonged to *Dermacentor andersoni*. *Coxiella burnetii* is acquired by arthropods during the hematophagy process; however, not all ticks that feed on infected animals become infected with this pathogen. In general, the acquisition of *C*. *burnetii* is harmless for ticks, in contrast to *Coxiella*-like endosymbionts, they are non-infectious for vertebrates, but they can be problematic for ticks, and this issue can be a major concern for conducting epidemiological studies to identify *C*. *burnetii* in ticks [37]. Based on the studies, it seems that ticks are not necessary for the transmission of *C*. *burnetii* to livestock, but among vertebrates, including rodents and wild birds, they probably play an important role. According to some studies, ticks seem to excrete a significant amount of these bacteria along with their faeces when they defecate, and inhalants of these infected particles by domestic animals can infect them [8, 38, 39]. Also, humans, while shearers, may get Q fever as a result of inhaling the particles of this bacterium [8]. The role of ticks in the transmission cycle of this bacterium has been discussed in many studies [40, 41]. DNA of *C*. *burnetii* has been detected in 40 species of ticks [8]. Furthermore, the presence of DNA of *C*. *burnetii* in *Ixodes*, *Hyalomma*, and *Rhipicephalus* ticks in the present study indicates the presence of this bacterium in a wide range of tick genera and shows the role of ticks in the epidemiology of Q fever [42, 43].

The present study indicated a significant relationship between *C*. *burnetii* infection and the genus of *Rhipicephalus*. The most common positive pools belonged to *Rh*. *sanguineus* sensu lato (26.1%), our results were similar to a study on ticks of dogs in Kerman province, and the results indicated a prevalence of 12.5% of *C*. *burnetii* in *Rh*. *sanguineus* sensu lato [44]. The first study in Iran on *C*. *burnetii* infection among ticks in 2009 indicated an 8.6% *C*. *burnetii* infection in *Hy*. *anatolicum* and *Rh*. *sanguineus*, with the highest belonging to *Hy*. *anatolicum* species [45]. In a study in Iran in 2020, 13.9% of ticks collected from sheep were reported positive for *C*. *burnetii* and the highest prevalence of infection was found in *D*. *marginatus* (18.3%) and *Ha*. *concinna* (12.5%) [46]. Also, in another study in Iran, 7.4% of ticks collected from sheep and goats belonging to *Rh*. *sanguineus* and *Hy*. *anatolicum* species were reported positive for *C*. *burnetii* [47]. In Cyprus, 6.4% of *Hyalomma* spp. and *Rh*. *sanguineus* ticks were positive, while most positive samples belonged to the *Rh*. *sanguineus*, consistent with the results obtained in the present study [48].

Real-time PCR did not detect *C*. *burnetii* infection in the genus *Haemaphysalis*. In a study in Turkey, the DNA of *C*. *burnetii* was detected in *Rhipicephalus* and *Hyalomma* genus, and most cases belonged to the *Rhipicephalus* genus, and it was consistent with our results, while there were not any positive samples in the genus of *Haemaphysalis* [49]. Like other studies and the results of our study, *Rhipicephalus* were the most common genera infected with *C*. *burnetii*. Other studies have also identified *C*. *burnetii* in *I*. *ricinus*. For example, in a study in Slovakia and Hungary, *C*. *burnetii* was detected in *I*. *ricinus*, *D*. *marginatus*, and *Ha*. *concinna* species [42]. According to a study in Germany on *I*. *ricinus*, 1.9% of the ticks were infected with *C*. *burnetii* [50]. In another study in Poland, 15.9% of *I*. *ricinus* collected from forest areas were infected with this pathogen [51]. In this study, the DNA of *C*. *burnetii* was also detected in *Ixodes* (3.1%). It seems likely that *I*. *ricinus* also play a role in the transmission cycle of *C*. *burnetii*.

There are few studies on *F*. *tularensis* infection in Iran, and most studies were conducted on human disease. In neighboring countries, few studies were performed on ticks as vectors of this disease [26]. DNA of *F*. *tularensis* was detected from ticks in all provinces, and the highest positive cases belonged to the Golestan province (10. 9%). However, there was no significant correlation between positive *F*. *tularensis* in ticks and the provinces.

According to the studies, the most common tick species in which the DNA of *F*. *tularensis* was detected belong to *Dermacentor*, *Haemaphysalis*, *Amblyomma*, and *Ixodes* genera, and they are very important in the terrestrial cycle of *F*. *tularensis* [52]. The most positive samples were identified in the *Haemaphysalis* genus and *Ha*. *inermis* species (100%). In a study in the UAE, the DNA of *F*. *tularensis* was identified in 5.8% of *Hy*. *dromedarii* isolated from camels by the molecular method [53]. In Egypt, 4.7% of *Hy*. *dromedarii* which were collected from camels were reported positive for *F*. *tularensis* [54]. However, none of the *Hy*. *dromedarii* collected from camel was reported positive for *F*. *tularensis* in our study.

In neighboring countries of Iran, including Turkey, *F*. *tularensis* is an endemic disease. However, according to the studies conducted on ticks in different sites in Turkey, no positive samples have been reported for *F*. *tularensis* [55–57]. While in our study, the DNA of *F*. *tularensis* was identified in 4 genera of ticks (*Ixodes*, *Haemaphysalis*, *Rhipicephalus*, and *Hyalomma*) collected from domestic animals using the molecular method.

According to some studies, ticks are important biological vectors for *F*. *tularensis*, which transmit this pathogen between humans and animals through bites and can maintain this organism for a long time in nature [58, 59]. *F*. *tularensis* can be localized in the gut and hemolymph of ticks, and the number of organisms increases from the larval stage to adult ticks [60]. However, some studies have suggested the possible role of ticks as a reservoir, which requires further investigation. Also, transstadial transmission of *F*. *tularensis* has been confirmed, but transmission through the transovarial is still debated [61]. Ticks also play a crucial role as biological and mechanical vectors for *F*. *tularensis*. Their life cycle involves four stages, namely egg, larva, nymph, and adult. Ticks require blood meals during their growth stages, making them capable of transmitting the bacteria through bites [62, 63].

Based on data from epidemiological studies, rodents, and rabbits have been suggested as the main reservoirs of *F*. *tularensis* [64]. Tularemia is often fatal in animals. Studies show that a bacteremia dose of more than 10^8^ CFU/ mL in the blood, spleen, and lung of a mic is a lethal dose. However, some rodents can survive without symptoms and transmit the infection to other rodents through ticks [52]. In a study in China to investigate *F*. *tularensis* in the spleen of rodents, 4.7% of the rodents were infected with this pathogen, but none of the samples were reported positive in our study [65].

According to a study in Turkey, the prevalence of *C*. *burnetii* and *F*. *tularensis* were reported at 40% and 22.5% in ticks, respectively. Based on the results of this study, most of the positive samples for *C*. *burnetii* were detected in questing ticks, which was consistent with our results (39.4%), while the positive samples for *F*. *tularensis* were detected in ticks collected from animals, which is not consistent with our results [66].

The number of rodents examined in this study was very limited and this was one of the limitations of our study. It is also possible that transient bacteremia in rodents prevented the detection of both pathogens investigated in this study. IS *1111* sequence is a specific transposon in *C*. *burnetii*, and it is widely used in the molecular diagnosis of infection by *C*. *burnetii* in humans and animals. Nevertheless, sometimes, similar IS *1111*- homolog is also seen in endosymbionts and *Coxiella*-likes bacteria in ticks, and it may cause a false positive result of *C*. *burnetii* in ticks. Unfortunately, we only used IS *1111* to screen ticks in this study, and it was recommended that another specific gene be used to confirm the IS *1111*-positive sample in future studies.

The positivity of ticks in the present study suggests a possibility of livestock infection, and because of the effects of these diseases on livestock, such as abortion, the possibility of human infection through livestock products, such as consuming contaminated milk, needs to be investigated. The results of this study increase our knowledge about the prevalence of these pathogens in Iran. The results also indicated that livestock ticks are infected with these pathogens, so more studies need to be conducted.

## Conclusions

It seems that ticks can be a reservoir and vector of pathogenic pathogens and are responsible for the transmission of infection to domestic and wild animals and even humans. The results of this study showed the contamination of ticks in domestic animals. Considering the possibility of animal contamination by ticks, the examination of animal samples is also useful to identify the prevalence of the above pathogens, because animals can be the mediators of disease transmission to humans. Because *C*. *burnetii* and *F*. *tularensis* significantly impact public health, it is very necessary to identify the main source of their spread and manage the situation. Therefore, it is necessary to fully investigate the role of ticks in the epidemiology of diseases and prepare a suitable educational program to prevent and control the population of ticks.
